# Supplementary material for: Divergent binding mode for a protozoan BRC repeat to RAD51
Source: Biochem J. 2022 May 23;479(10):1031–43. doi: 10.1042/BCJ20220141 (PMC9162458; doi:10.1042/BCJ20220141)
Supplement: Supplementary Material [file BCJ-479-1031-s1.pdf]

SUPPLEMENTARY DATA

DIVERGENT BINDING MODE FOR A PROTOZOAN BRC REPEAT TO RAD51

Teodors Pantelejevs[1,2*] and Marko Hyvönen[1*]

[1] Department of Biochemistry, University of Cambridge, CB2 1GA, UK

[2] Present address: Latvian Institute of Organic Synthesis, Aizkraukles 21, Riga, LV-1006, Latvia

**Protein**	0.5 mM *Li*BRC1 :*Li*RAD51$^{ATPase,\Delta L2}$ in 20 mM Tris pH 8.0, 100 mM NaCl, 100 mM Li2SO4, 20 mM ADP/$MgCl_2$
**Condition**	32% low MW PEG smear (precipitant, Molecular Dimensions), 0.1M Tris pH 8.5
**Protein:condition volume (nl:nl)**	200:200
**PDB**	7QV8
**Data collection processing**	
Beamline	DLS i04-1
Wavelength (Å)	0.9159
Space group	P 4 2 2
a, b, c (Å)	61.00, 61.00, 119.22
α, β, γ (°)	90.00, 90.00, 90.00
Resolution range (high-resolution bin) (Å)	2.15 – 59.61 (2.15 – 2.18)
Rmeas	0.119 (8.167)
Completeness (%)	98.9 (98.9)
Number of total / unique reflections	296871 / 12826
Redundancy	23.1 (18.1)
$\langle I/\sigma(I) \rangle$	15.6 (0.4)
CC1/2	1.0 (0.5)
**Refinement**	
R_{cryst} /R_{free}	0.211 / 0.267
Resolution range (Å)	54.31 – 2.15
Number of reflections: work/test set	10597 / 517
Number of atoms	1806
Mean/Wilson B-factor	67.897 / 50.7
Ramachandran favoured/allowed/outliers (%)	98.65 / 1.35 / 0.00
RMSD bonds (Å)	0.012
RMSD angles (°)	1.62

Table S1. Crystallisation conditions, data collection and refinement statistics. Values in parentheses are for the high-resolution bin.

Insert	Vector	Res. Enzyme(s)	No	Sequence
*Li*BRC1	pPEPT1	*BsaI*	1	TTCCAGGGATCCGACCCTCTGGTTCCGACCCTGTTCTCTACCGCGTCTGGTAAACCGGTGACCGTTCGTCGTG
			2	ATGGCTCGAGCCAGGGTCCGCCGCCAGGTCACCCAGACGTTCCGCAACTTTCTGCAGGGATTCACGACGAACGGTCACC
	pOP3BT	*BamHI/HindIII*	1	GAAAACCTGTACTTC CAGGGATCCCTGGTTCCGACCCTGTTCT
			2	ACGAACGGTCACCGGTTTACCAGACGCGGTAGAGAACAGGGTCGGAACCA
			3	AACCGGTGACCGTTCGTCGTGAATCCCTGCAGAAAGTTGCGGAACGTCTG
			4	GGTGACACTATAGAATACTCAAGCTTAGGCGGCCAAGTCACCCAGACGTTCCGCAACTTTCT
Ncys-*Li*BRC1	pOP3BT	*BamHI/HindIII*	1	AACCTGTACTTCCAGGGATCCTGCCGTGTTCCGACCCTGTTC
			2	TGAACGGTAACCGCTTTACCACGACCGGTTTCGAACAGGGTCGGAACACG
			3	GGTAAAGCGGTTACCGTTCAGAAACGTTCTCTGGACAAAGCGGAAGCGTC
			4	ACTATAGAATACTCAAGCTTAGGCTTCTAAAGAGTCCATAGACGCTTCCGCTTTGTCC
*Li*BRC1.1	pPEPT1	*BsaI*	1	TTCCAGGGATCCGACCCTCTGGTTCCGACCCTGTTCTCTACCGGTTCTGGTAAACCGGTGACCGTTCGTCGTG
			2	ATGGCTCGAGCCAGGGTCCGCCGCCAGGTCACCCAGACGTTCCGCAACTTTCTGCAGGGATTCACGACGAACGGTCACC
*Li*BRC1.2	pPEPT1	*BsaI*	1	TTCCAGGGATCCGACCCTCTGGTTCCGACCCTGTTCTCTACCGCGTCTGGTAAGCCGGTTACCGTTCGTCGTGAATCTCTC
			2	ATGGCTCGAGCCAGGGTCTTCCATATCAGGTGCGGCCAGGTCACCCAGACGTTCCGCAACTTTCTGGAGAGATTCACGACGAACGG
*Li*BRC1.3	pPEPT1	*BsaI*	1	TTCCAGGGATCCGACCCTGCTGCGGCTCCGCCGCTGGTTCCGACCCTGTTCTCTACCGCGTCTGGTAAGCCGGTTACCGTTCG
			2	ATGGCTCGAGCCAGGGTCCGCCGCCAGGTCACCCAGACGTTCCGCAACCTTTTGCAGAGATTCACGACGAACGGTAACCGGCTTAC
*Li*BRC1.4	pPEPT1	*BsaI*	1	TTCCAGGGATCCGACCCTCTGGTTCCGACCCTGTTCTCTACCGCGTCTGGTAAACCGGTGACCGTTC
			2	ATGGCTCGAGCCAGGGTCGTCACCCAGACGTTCCGCAACTTTCTGCAGGGATTCACGACGAACGGTCACCGGTTTACC
*Li*BRC1.5	pPEPT1	*BsaI*	1	TTCCAGGGATCCGACCCTCTGGTTCCGACCCTGTTCTCTACCGCGTCTGGTAAACCGGTGACCGTTCGTCGTG
			2	ATGGCTCGAGCCAGGGTCAGAACCACCACCCAGACGTTCCGCAACTTTCTGCAGGGATTCACGACGAACGGTCACCG
*Li*BRC1.6	pPEPT1	*BsaI*	1	TTCCAGGGATCCGACCCTCTGGTTCCGACCCTGTTCTCTACCGCGTCTGGTAAACCGGTGACCGTTCGTCGTG
			2	ATGGCTCGAGCCAGGGTCAGAGCCACCACCACGTTCCGCAACCTTCTGCAGGGATTCACGACGAACGGTCACCGGTT
*Li*BRC1.7	pPEPT1	*BsaI*	1	TTCCAGGGATCCGACCCTGCTGCGGCTCCGCCGCTGGTTCCGACCCTGTTCTCTACCGCGTCTGGTAAGCCGGTTACCGTTCG
			2	ATGGCTCGAGCCAGGGTCCGCCGCCAGGTCACCCAGACGTTCCGCAACCTTTTGCAGAGATTCACGACGAACGGTAACCGGCTTAC
*Li*BRC1.8	pPEPT1	*BsaI*	1	TTCCAGGGATCCGACCCTCTGGTTCCGACCCTGTTCTCTACCGCGTCTGGTAAGCCGGTTACCGTTCGT
			2	ATGGCTCGAGCCAGGGTCAGAGCCACCGCCACCCGCAACTTTCTGCAGGGATTCACGACGAACGGTAACCGGCTTACC
*Li*BRC1.9	pPEPT1	*BsaI*	1	TTCCAGGGATCCGACCCTCTGGTTCCGACCCTGTTCTCTACCGCGTCTGGTAAGCCGGTTACCGTTCGT
			2	ATGGCTCGAGCCAGGGTCAGAGCCACCGCCACCAACTTTCTGGAGAGATTCACGACGAACGGTAACCGGCTTACCAG
*Li*BRC1.10	pPEPT1	*BsaI*	1	TTCCAGGGATCCGACCCTCTGGTTCCGACCCTGTTCT
			2	ACGAACGGTCACCGGTTTACCAGACGCGGTAGAGAACAGGGTCGGAACCA
			3	AACCGGTGACCGTTCGTCGTGAATCTCTCCAGAAAGGTGGCTCTGGTGGT
			4	ATGGCTCGAGCCAGGGTCGGAACCACCAGAGCCACCTTT
*Li*BRC1.11	pPEPT1	*BsaI*	1	TTCCAGGGATCCGACCCTCTGGTTCCGACCCTGTTCT
			2	ACGAACGGTCACCGGTTTACCAGACGCGGTAGAGAACAGGGTCGGAACCA
			3	AACCGGTGACCGTTCGTCGTGAATCTCTCCAGGGTGGCTCTGGTGGTTCC
			4	ATGGCTCGAGCCAGGGTCGGAACCACCAGAGCCACC
*Li*BRC1.12	pPEPT1	*BsaI*	1	TTCCAGGGATCCGACCCTCTGGTTCCGACCCTGTTCTCTACCGCG
			2	ATGGCTCGAGCCAGGGTCACGAACGGTTACCGGTTTACCAGACGCGGTAGAGAACAGGGT
*Li*BRC2	pPEPT1	*BsaI*	1	TTCCAGGGATCCGACCCTCGTGTTCCGACCCTGTTCGAAACCGGTCGTGGTAAAGCGGTTACCGTTCAGAAACGTTC
			2	ATGGCTCGAGCCAGGGTCCGCTTCCAGAGAGTCCATAGACGCTTCCGCTTTGTCGAGAGAACGTTTCTGAACGGTAACC
*Li*BRC2.1	pPEPT1	*BsaI*	1	TTCCAGGGATCCGACCCTCGTGTTCCGACCCTGTTCGAAACCGCGCGTGGTAAAGCGGTTACCGTTCAGAAACGTTC
			2	ATGGCTCGAGCCAGGGTCCGCTTCCAGAGAGTCCATAGACGCTTCCGCTTTGTCGAGAGAACGTTTCTGAACGGTAACC
*Li*RAD51 (full-length)	pEXP-MBP	*BsaI/HindII*	1	GAAAACCTGTACTTCCAGTCCGGACAGACCCGTAGCAAAGC
			2	ATAGAATACTCAAGCTTATTAGTCACGTGCGTCACCAAC
*Li*RAD51^{ATPase}	pHAT2	*NcoI/HindIII*	1	CCATCACCATCACTCCATGGCAGAAATTATCATGGTTACCACCGGTAG
			2	ACTATAGAATACTCAAGCTTAGTCACGTGCGTC
*Li*RAD51$^{ATPase,\Delta L2}$	pBAT4	*NcoI/HindIII*	1	AAGGAGATATATCCATGGCAGAAATTATCATGGTTACCACCGGTAG
			2	ACTATAGAATACTCAAGCTTAGTCACGTGCGTC
			3	GTTGTTGCCAATGGTGGTCATATTATGGCACATGCCAGC (inner F)
			4	CATAATATGACCACCATTGGCAACAACCTGATTGGTAACAACAAC (inner R)

Table S2. Oligonucleotides used for assembly PCR and cloning.

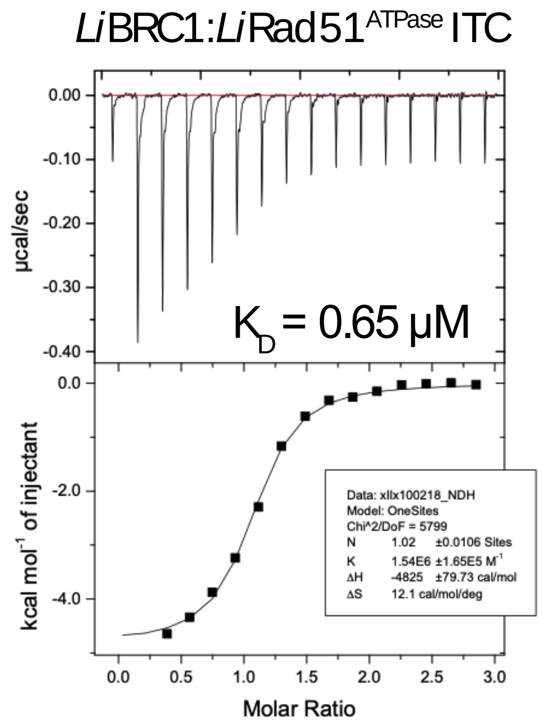

Figure S1. ITC titration of *Li*BRC1 peptide into *Li*RAD51^{ATPase}.

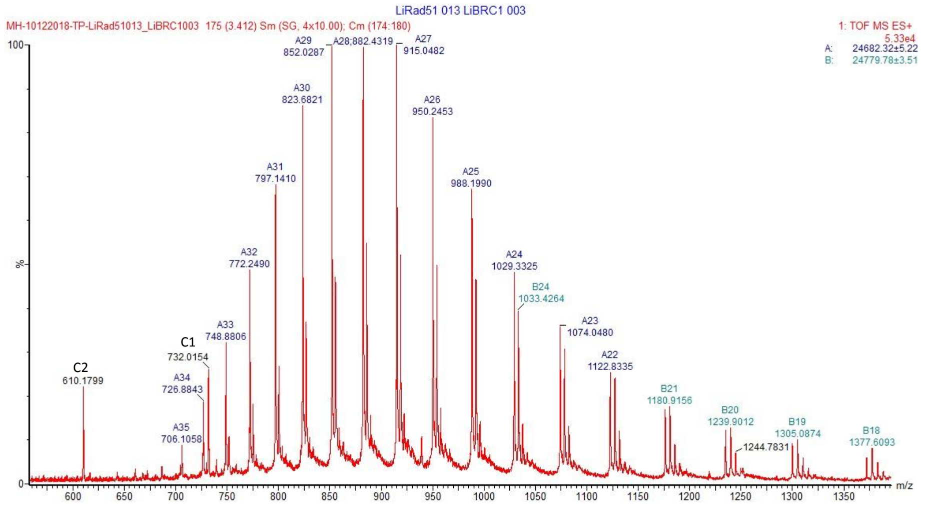

Species	m/z calculated	m/z found
M+5H (C1)	732.04	732.02
M+6H (C2)	610.20	610.18

Figure S2. Protein mass spectrum of the *Li*BRC1:*Li*RAD51$^{ATPase,\Delta L2}$ complex. Peaks C1 and C2 correspond to the full-length *Li*BRC1 peptide.

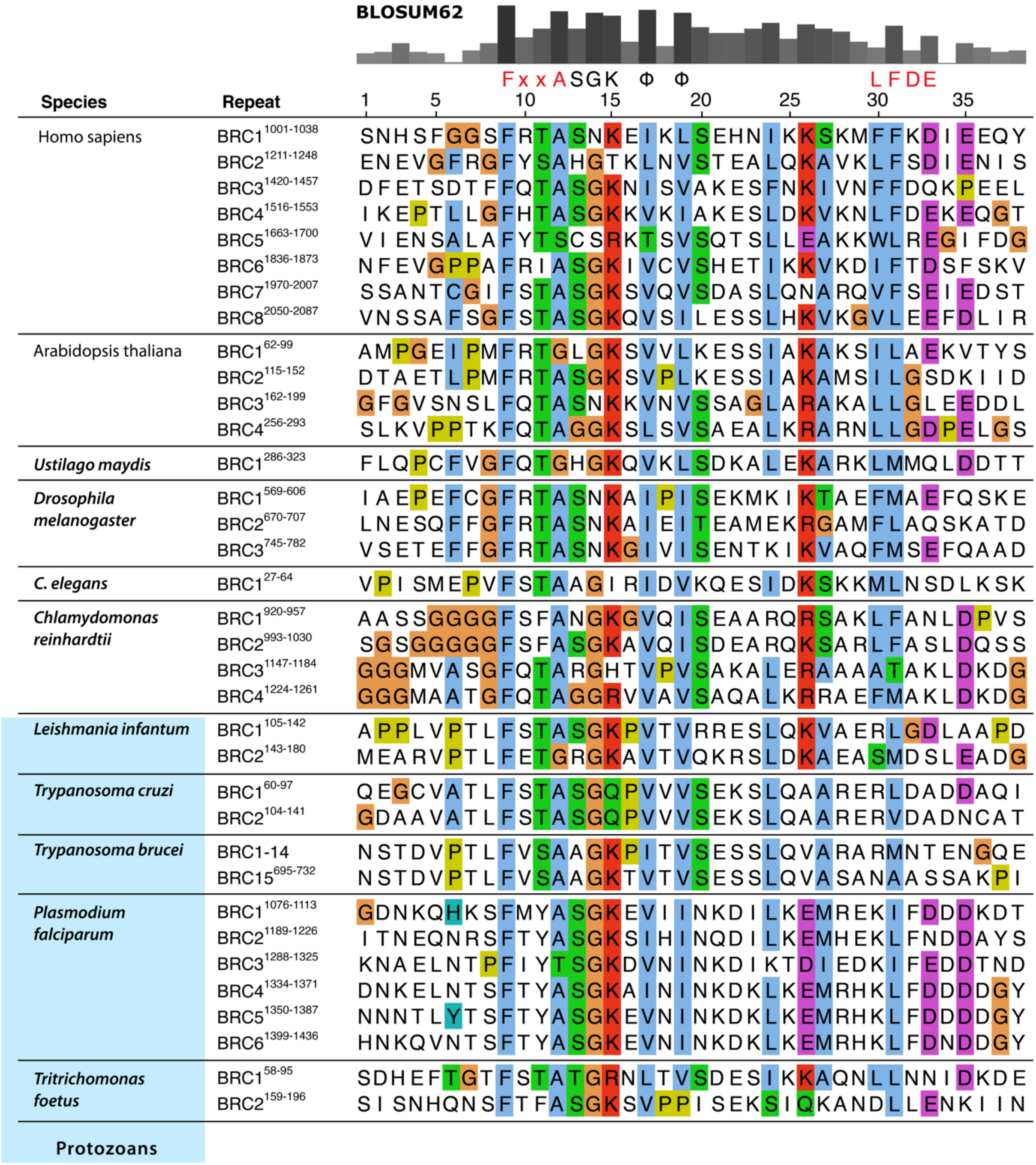

Figure S3. Sequence alignment of BRC repeats from a set of representative organisms and protozoan parasites. The grey bars on top represent BLOSUM62 alignment scores. Conserved residues are coloured using the default ClustalX colour scheme. BRC1-14 in *T. brucei* refers to the identical set of 14 repeats, BRC1-BRC14.

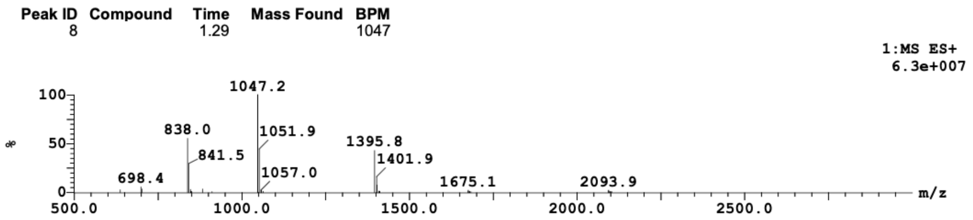

Species	m/z calculated	m/z found
M+3H	1396.24	1395.8
M+4H	1047.43	1047.2
M+5H	838.15	838.0
M+6H	700.02	698.4

Figure S4. Mass spectrum of the *Li*BRC1-fluor peptide.
